# Comparison of Antibiotic Prophylaxis Regimens for Preventing Prostatitis After Transrectal Ultrasound-Guided Prostate Biopsy

**DOI:** 10.7759/cureus.114982

**Published:** 2026-08-22

**Authors:** Mohamad Jaber, Alaa Jazzar, Mohamad Kbar, Mohammad Ajourch, Walid Alameh

**Affiliations:** 1 Urology, Lebanese University Faculty of Medical Sciences, Hadath, LBN; 2 Urology, Bahman Hospital, Beirut, LBN; 3 Urology, Sahel General Hospital, Beirut, LBN

**Keywords:** amikacin, ertapenem, fluoroquinolones, prostatitis, trus-guided biopsy

## Abstract

Background: Transrectal ultrasound (TRUS)-guided prostate biopsy is a common way to find prostate cancer, but it can lead to infections, including sepsis. Fluoroquinolones have historically served as a means of antibiotic prophylaxis; however, escalating resistance has diminished their efficacy, thereby requiring alternative prophylactic approaches.

Objective: To compare infectious outcomes after TRUS-guided prostate biopsy across various antibiotic prophylaxis regimens and to evaluate the efficacy of single-dose intramuscular (IM) ertapenem and amikacin.

Methods: This retrospective observational study encompassed patients who underwent TRUS-guided prostate biopsy from January 2015 to June 2025 at two hospitals in Lebanon. We looked at the patients' demographics, clinical characteristics, laboratory data, antibiotic prophylaxis regimens, and post-biopsy infectious complications. We used bivariate analysis and multivariable logistic regression to find links between prophylaxis strategies and the risk of infection.

Results: There were 209 patients in total, with an average age of 66.7 years and an average prostate-specific antigen level of 18.2 ng/mL. In total, 12.9% of patients had infections after the biopsy. Patients who received single-dose IM ertapenem, either alone or in combination with fluoroquinolones, did not experience any infections. The infection rate for patients who got a single dose of IM amikacin was 3.8%, while the rate for patients who got fluoroquinolone monotherapy was 26%. Single-dose IM ertapenem or amikacin was linked to a lower risk of infection than fluoroquinolone-based prophylaxis (p < 0.05). There was no meaningful distinction between ertapenem and amikacin, nor between single-agent and combination regimens. Older age and a history of positive urine culture were strongly linked to post-biopsy infection.

Conclusion: A single dose of IM ertapenem or amikacin appears to be associated with a lower risk of infections after a TRUS-guided prostate biopsy. They may represent a potential prophylactic alternative to fluoroquinolone-based regimens, especially in places where routine rectal culture-guided prophylaxis is not done.

## Introduction

Prostate cancer is the second most commonly diagnosed cancer among men worldwide and represents the most common cancer in males in Lebanon, accounting for 16.4% of all cancers diagnosed in men [1]. Strategies for early detection of prostate cancer have evolved significantly over time. Historically, digital rectal examination (DRE) was the primary clinical tool used to identify patients with suspected prostate cancer who required further evaluation. Over the past few decades, the widespread use of prostate-specific antigen (PSA) testing has significantly increased the detection of prostate cancer at earlier stages. Additional diagnostic investigations are usually considered when serum PSA levels reach or exceed 4 ng/mL, as patients with PSA levels between 4 and 10 ng/mL have an estimated prostate cancer detection risk of 20-30% [2].

Prostate biopsy remains the gold standard for the definitive diagnosis of prostate cancer. Several biopsy techniques have been developed over time. Initially, finger-guided transrectal biopsy was used, followed by transrectal ultrasound (TRUS)-guided biopsy, which provided improved accuracy and anatomical guidance. Despite their widespread use, transrectal biopsy techniques are associated with an infectious complication rate of approximately 3%. More recently, transperineal ultrasound-guided biopsy has emerged as an alternative approach, with a lower risk of infection and improved detection of anterior prostate lesions [3].

Although the transperineal approach has demonstrated advantages regarding infection prevention, TRUS-guided prostate biopsy remains widely performed worldwide. Due to the risk of infectious complications, including potentially life-threatening sepsis, antibiotic prophylaxis remains an essential component of patient management before this procedure [4]. Various antibiotic regimens have been used for infection prevention, with fluoroquinolones historically being the most commonly prescribed agents due to their demonstrated effectiveness in reducing post-biopsy infections [5]. However, increasing resistance to fluoroquinolones has significantly reduced their efficacy, with resistance rates reaching approximately 20% among rectal flora cultures [6].

The increasing prevalence of fluoroquinolone-resistant organisms has encouraged the investigation of alternative prophylactic strategies. Fosfomycin trometamol has been evaluated as a potential alternative and has demonstrated a lower risk of post-biopsy infectious complications [7]. Furthermore, a single-dose regimen of ertapenem has been shown to significantly reduce infectious complications following transrectal prostate biopsy compared with a three-day fluoroquinolone regimen [8]. The use of pre-biopsy rectal cultures has also allowed targeted antibiotic prophylaxis, with studies demonstrating that a single 1 g dose of ertapenem provides effective coverage against fluoroquinolone-resistant organisms [9]. Additionally, combining aminoglycosides, such as amikacin, with fluoroquinolone-based prophylaxis has demonstrated effectiveness in reducing post-biopsy infection rates [10].

Given the increasing prevalence of antibiotic-resistant organisms and the significant variation in prophylactic regimens used in clinical practice, further studies are needed to identify effective prophylactic strategies while minimizing the risk of antimicrobial resistance. Therefore, this study aimed to compare the risk of infectious complications following TRUS-guided prostate biopsy among patients receiving different antibiotic prophylaxis regimens. Additionally, we aimed to evaluate the effectiveness of ertapenem and amikacin in preventing post-biopsy infections and to compare single-agent prophylaxis with combination antibiotic regimens.

## Materials and methods

Study design, location, and data acquisition

This was a retrospective observational study that included patients who underwent TRUS-guided prostate biopsy between January 2015 and June 2025. Data were collected from the medical records of patients who underwent the procedure during the study period. The collected variables included patient age, comorbidities, history of previous infections, pre-biopsy urine culture results, PSA levels, prostate volume, type of antibiotic prophylaxis administered, and post-biopsy infectious outcomes.

Post-biopsy infection was defined as the presence of fever ≥38°C associated with urinary symptoms and/or a positive urine culture, or hospital admission for sepsis within 30 days following the biopsy procedure. The choice of antibiotic prophylaxis regimen was based on the treating physician’s preference, and no standardized risk stratification protocol was applied before biopsy.

Patients were recruited from two hospitals in Lebanon: Bahman Hospital and Sahel General Hospital. To ensure the accuracy and completeness of the collected data, patients were contacted by telephone to obtain informed consent and to confirm the clinical information extracted from their medical records.

Study population

Patients who underwent TRUS-guided prostate biopsy between January 2015 and June 2025 and received at least one antibiotic agent as prophylaxis before the procedure were eligible for inclusion.

Patients were excluded if they had incomplete or missing medical records, underwent transperineal prostate biopsy, were immunocompromised, or were chronic catheter users.

Ethical considerations

All patients were contacted by telephone to obtain informed consent before the use of their medical information for research purposes. Ethical approval was obtained from the Institutional Review Boards (IRBs) of both Sahel General Hospital and Bahman Hospital before the initiation of the study.

Statistical analysis

Statistical analysis was performed using SPSS version 24 (IBM Corp., Armonk, NY). Descriptive statistics were used to summarize patient characteristics, including age, comorbidities, PSA levels, prostate volume, antibiotic prophylaxis regimens, and the incidence of post-biopsy infectious complications.

Continuous variables were presented as mean ± standard deviation (SD), while categorical variables were presented as number (N) and percentage (%). Comparisons between antibiotic prophylaxis groups and assessment of associations between categorical variables and post-biopsy infection risk were performed using the chi-square test or Fisher’s exact test when appropriate.

Binary logistic regression analysis was performed to evaluate the association between patient-related factors, including age, prostate volume, and PSA level, and the risk of post-biopsy infection. Results from logistic regression analysis were presented as odds ratios (ORs) with 95% confidence intervals (CIs). Relative risks (RRs) with 95% CIs were calculated for bivariate comparisons between antibiotic prophylaxis groups. A two-sided p-value <0.05 was considered statistically significant.

## Results

A total of 209 patients were included in the study. The mean age of the study population was 66.69 ± 8.817 years. The mean PSA level was 18.24 ± 22.59 ng/mL, with a mode of 7 ng/mL, and the mean prostate volume was 49.6 ± 24.5 g (Table 1). Regarding comorbidities, hypertension was present in 128 (61.2%) patients, diabetes mellitus in 50 (23.9%) patients, and coronary artery disease in 39 (18.7%) patients. A positive urine culture within the preceding 12 months was documented in 11 (5.3%) patients. Prostate cancer was detected following biopsy in 86 (41.1%) patients (Table 2).

**Table 1:** The mean age, prostate size, and PSA of the patients. Data are presented as mean values. PSA: prostate-specific antigen.

Variable	Mean ± SD
Age	66.69 ± 8.817 years
Prostate size	49.6 ± 24.5 g
PSA	18.24 ± 22.59 ng/ml

**Table 2:** Clinical and laboratory characteristics of the patients. Data are presented as numbers (percentages), N (%). Statistical analysis of categorical variables was performed using the chi-square test or Fisher’s exact test when appropriate. A p-value <0.05 was considered statistically significant.

Clinical and laboratory characteristics	Yes, N (%)	No, N (%)
Hypertension	128 (61.2%)	81 (38.8%)
Diabetes mellitus	50 (23.9%)	159 (76.1%)
Coronary artery disease	39 (18.7%)	170 (81.3%)
Positive urine culture within the previous 12 months	11 (5.3%)	198 (94.7%)
Prostate cancer detected after biopsy	86 (41.1%)	123 (58.9%)

Regarding antibiotic prophylaxis, 15 patients received a single intramuscular (IM) dose of ertapenem, and none developed post-biopsy infection. A total of 26 patients received a single IM dose of amikacin, among whom one (3.8%) patient developed an infectious complication. Ninety-six patients received oral fluoroquinolone prophylaxis (prulifloxacin, ciprofloxacin, or levofloxacin) for one week, and 25 (26%) patients developed post-biopsy infection. Forty-seven patients received a single IM dose of ertapenem, followed by one week of oral fluoroquinolones, with no infectious complications reported. Twenty-five patients received a single IM dose of amikacin, followed by one week of oral fluoroquinolones, and one (4%) patient developed an infection. Overall, post-biopsy infectious complications occurred in 27 (12.9%) of the 209 included patients (Table 3).

**Table 3:** The distribution of different antibiotic regimens among patients. Data are presented as numbers (percentages), N (%). Antibiotic groups were compared using Fisher’s exact test or the chi-square test when appropriate. A p-value <0.05 was considered statistically significant.

Antibiotic prophylaxis regimen	No infection, N (%)	Infection, N (%)	Total, N (%)	Infection rate
Ertapenem only	15 (100%)	0 (0%)	15 (7.2%)	0%
Amikacin only	25 (96.2%)	1 (3.8%)	26 (12.4%)	3.8%
Fluoroquinolones	71 (74%)	25 (26%)	96 (45.9%)	26%
Ertapenem + fluoroquinolones	47 (100%)	0 (0%)	47 (22.5%)	0%
Amikacin + fluoroquinolones	24 (96%)	1 (4%)	25 (12%)	4%
Total	182 (87.1%)	27 (12.9%)	209 (100%)	12.9%

In the comparison between antibiotic prophylaxis regimens, no statistically significant difference was observed between a single IM dose of ertapenem and a single IM dose of amikacin (RR: 0.56, 95% CI: 0.02-12.9, p > 0.05). Similarly, no significant difference was found between amikacin alone and amikacin combined with one week of oral fluoroquinolones (RR: 1.02, 95% CI: 0.248-4.191, p > 0.05).

However, compared with fluoroquinolone monotherapy, a single IM dose of ertapenem was associated with a lower risk of post-biopsy infection (RR: 0.208, 95% CI: 0.143-0.267, p = 0.016). Similarly, a single IM dose of amikacin was associated with a reduced risk of infection compared with fluoroquinolone prophylaxis (RR: 0.148, 95% CI: 0.104-0.579, p = 0.014) (Table 4).

**Table 4:** Difference in the effect of antibiotics prophylaxis on the risk of infection. Relative risks (RRs) with 95% confidence intervals (CIs) were calculated for comparisons between antibiotic prophylaxis groups. Statistical analysis was performed using Fisher’s exact test due to small subgroup sizes. A p-value <0.05 was considered statistically significant.

Antibiotic comparison	Relative risk (RR), 95% CI	p-value
Ertapenem vs. amikacin	0.56 (0.02-12.9)	>0.05
Ertapenem vs. fluoroquinolones	0.208 (0.143-0.267)	0.016
Amikacin vs. amikacin + fluoroquinolones	1.02 (0.248-4.191)	>0.05
Amikacin vs. fluoroquinolones	0.148 (0.104-0.579)	0.014

Regarding patient-related factors, increasing age and a history of positive urine culture within the previous 12 months were significantly associated with an increased risk of post-biopsy infection. Patients who developed infection had a higher mean age compared with those without infection (70.15 vs. 66.18 years; OR: 1.057, 95% CI: 1.008-1.110, p = 0.023). Prostate volume and PSA level were not significantly associated with infection risk (Table 5).

**Table 5:** The effect of age, prostate size, and PSA on the risk of infection. Data are presented as mean values. Odds ratios (ORs) with 95% confidence intervals (CIs) were obtained from binary logistic regression analysis. A p-value <0.05 was considered statistically significant. PSA: prostate-specific antigen.

Variable	No infection (mean)	Infection (mean)	OR (95% CI)	p-value
Age (years)	66.18	70.15	1.057 (1.008-1.110)	0.023
Prostate volume (g)	49.7	49.0	0.996 (0.978-1.014)	0.649
PSA level (ng/mL)	18.5	16.5	0.996 (0.978-1.014)	0.643

Among comorbidities, hypertension, diabetes mellitus, and coronary artery disease were not significantly associated with post-biopsy infection. In contrast, patients with a previous positive urine culture had a significantly higher risk of infection after biopsy (RR: 1.32, 95% CI: 1.055-1.652, p < 0.001) (Table 6).

**Table 6:** The effect of comorbidities and previous positive culture on the risk of infection. Data are presented as numbers (percentages), N (%). Relative risks (RRs) with 95% confidence intervals (CIs) were calculated using chi-square or Fisher’s exact test when appropriate. A p-value <0.05 was considered statistically significant.

Variable	No infection, N (%)	Infection, N (%)	RR (95% CI)	p-value
Hypertension	111 (85.9%)	17 (14.1%)	0.790 (0.373-1.673)	0.673
Diabetes mellitus	40 (80%)	10 (20%)	0.535 (0.262-1.091)	0.095
Coronary artery disease	31 (79.5%)	8 (20.5%)	0.545 (0.258-1.153)	0.182
Positive urine culture within the previous 12 months	4 (36.4%)	7 (63.6%)	1.32 (1.055-1.652)	<0.001

## Discussion

This study evaluated different antibiotic prophylaxis regimens used before TRUS-guided prostate biopsy and their association with post-biopsy infectious complications. Our findings demonstrated that a single IM dose of ertapenem was associated with a lower risk of post-biopsy infection. None of the patients who received IM ertapenem, either as monotherapy or in combination with oral fluoroquinolones, developed infectious complications following biopsy. Furthermore, ertapenem demonstrated better effectiveness compared with fluoroquinolone monotherapy. These findings are consistent with previous studies reporting low rates of infectious complications among patients receiving ertapenem prophylaxis before transrectal prostate biopsy [11]. Similarly, another study demonstrated that a single IM dose of ertapenem significantly reduced the incidence of post-biopsy infection and sepsis compared with prophylaxis using fluoroquinolones combined with amoxicillin-clavulanate [12].

Our findings also showed that a single IM dose of amikacin was effective in preventing post-biopsy infections and demonstrated comparable effectiveness to single-dose ertapenem. Moreover, the addition of one week of oral fluoroquinolones to amikacin prophylaxis did not provide additional benefit in reducing infection risk. To our knowledge, limited data are available regarding the use of amikacin as a single-agent prophylaxis regimen before TRUS-guided prostate biopsy. However, previous studies have demonstrated that adding amikacin to fluoroquinolone-based prophylaxis can reduce the incidence of septic complications compared with fluoroquinolone monotherapy [13]. Additionally, a systematic review reported that augmented prophylaxis, including the addition of aminoglycosides such as gentamicin or amikacin to fluoroquinolone regimens, was more effective in reducing post-biopsy infectious complications [14].

The increasing prevalence of fluoroquinolone-resistant organisms has led to the development of alternative prophylactic strategies. Some institutions recommend performing pre-biopsy rectal cultures to identify fluoroquinolone-resistant organisms or extended-spectrum beta-lactamase (ESBL)-producing bacteria and subsequently provide targeted antibiotic prophylaxis. This approach may reduce unnecessary broad-spectrum antibiotic use and potentially limit the development of antimicrobial resistance [9]. However, previous studies have suggested that administering a single 1 g dose of ertapenem before prostate biopsy does not significantly increase the risk of developing carbapenem-resistant organisms in rectal flora [15].

In the current study, a history of positive urine culture within the previous 12 months was significantly associated with an increased risk of post-biopsy infection. This finding suggests that previous urinary tract infections and recent antibiotic exposure may represent important risk factors for infectious complications after prostate biopsy. These results are consistent with previous systematic reviews reporting higher infection rates among patients with previous urinary tract infections or recent antibiotic exposure. Although some studies have identified diabetes mellitus and other comorbidities as potential risk factors for post-biopsy infections, our study did not demonstrate a significant association between diabetes, hypertension, or coronary artery disease and infection risk [16].

This study provides additional evidence supporting the potential role of ertapenem and amikacin as alternative prophylactic agents for preventing infectious complications after TRUS-guided prostate biopsy. A single-dose, single-agent regimen may provide adequate prophylaxis and appears to be more effective than fluoroquinolone-based prophylaxis in settings with increasing fluoroquinolone resistance.

However, several limitations should be considered when interpreting these results. First, the retrospective design of the study may introduce selection bias and limits the ability to establish causal relationships. In addition, recall bias may have occurred because some data were obtained from patients’ recollection of events over a period spanning the previous 10 years. Second, the relatively small sample size, particularly within the ertapenem-only and amikacin-only groups (15 and 26 patients, respectively), resulted in wide confidence intervals and limited the statistical power of subgroup comparisons. Additionally, the absence of infections in the ertapenem-only group makes interpretation difficult and prevents definitive conclusions regarding its true effectiveness. Finally, this study did not evaluate the impact of different prophylactic strategies on the development of antimicrobial resistance, particularly carbapenem-resistant organisms, which remains an important consideration when using broad-spectrum antibiotics [15].

## Conclusions

TRUS-guided prostate biopsy is still a common way to find prostate cancer, although it does come with a higher chance of getting infections after the procedure, such as sepsis. A single-dose injectable ertapenem or amikacin may represent a potential prophylactic alternative to fluoroquinolone-based prophylaxis for the prevention of post-biopsy infections, especially in contexts where rectal culture-guided targeted prophylaxis is not commonly practiced.

## References

[1] Lakkis NA, Osman MH (2021). Prostate cancer in Lebanon: incidence, temporal trends, and comparison to countries from different regions in the world. Cancer Control.

[2] Matlaga BR, Eskew LA, McCullough DL (2003). Prostate biopsy: indications and technique. J Urol.

[3] Omer A, Lamb AD (2019). Optimizing prostate biopsy techniques. Curr Opin Urol.

[4] Shigehara K, Miyagi T, Nakashima T, Shimamura M (2008). Acute bacterial prostatitis after transrectal prostate needle biopsy: clinical analysis. J Infect Chemother.

[5] Sieber PR, Rommel FM, Agusta VE, Breslin JA, Huffnagle HW, Harpster LE (1997). Antibiotic prophylaxis in ultrasound guided transrectal prostate biopsy. J Urol.

[6] Liss MA, Johnson JR, Carmody BJ (2012). Fluoroquinolone-resistant rectal colonization predicts risk of infectious complications after transrectal prostate biopsy. J Urol.

[7] Pilatz A, Dimitropoulos K, Veeratterapillay R (2020). Antibiotic prophylaxis for the prevention of infectious complications following prostate biopsy: a systematic review and meta-analysis. J Urol.

[8] Seitz M, Stief C, Waidelich R, Bader M, Tilki D (2017). Transrectal ultrasound guided prostate biopsy in the era of increasing fluoroquinolone resistance: prophylaxis with single-dose ertapenem. World J Urol.

[9] Liss MA, Taylor SA, Batura D (2014). Fluoroquinolone resistant rectal colonization predicts risk of infectious complications after transrectal prostate biopsy. J Urol.

[10] Batura D, Rao GG, Bo Nielsen P, Charlett A (2011). Adding amikacin to fluoroquinolone-based antimicrobial prophylaxis reduces prostate biopsy infection rates. BJU Int.

[11] Losco G, Studd R, Blackmore T (2014). Ertapenem prophylaxis reduces sepsis after transrectal biopsy of the prostate. BJU Int.

[12] Bloomfield MG, Wilson AD, Studd RC, Blackmore TK (2020). Highly effective prophylaxis with ertapenem for transrectal ultrasound-guided prostate biopsy: effects on overall antibiotic use and inpatient hospital exposure. J Hosp Infect.

[13] Kehinde EO, Al-Maghrebi M, Sheikh M, Anim JT (2013). Combined ciprofloxacin and amikacin prophylaxis in the prevention of septicemia after transrectal ultrasound guided biopsy of the prostate. J Urol.

[14] Yang L, Tang Z, Gao L (2016). The augmented prophylactic antibiotic could be more efficacious in patients undergoing transrectal prostate biopsy: a systematic review and meta-analysis. Int Urol Nephrol.

[15] Bloomfield MG, Page MJ, McLachlan AG, Studd RC, Blackmore TK (2017). Routine ertapenem prophylaxis for transrectal ultrasound guided prostate biopsy does not select for carbapenem resistant organisms: a prospective cohort study. J Urol.

[16] Roberts MJ, Bennett HY, Harris PN, Holmes M, Grummet J, Naber K, Wagenlehner FM (2017). Prostate biopsy-related infection: a systematic review of risk factors, prevention strategies, and management approaches. Urology.

